# The adverse impact of a gain in chromosome 1q on the prognosis of multiple myeloma treated with bortezomib-based regimens: A retrospective single-center study in China

**DOI:** 10.3389/fonc.2022.1084683

**Published:** 2022-12-20

**Authors:** Qingxiao Chen, Xiaoyan Han, Gaofeng Zheng, Yang Yang, Yi Li, Enfan Zhang, Li Yang, Mengmeng Dong, Donghua He, Jingsong He, Zhen Cai

**Affiliations:** ^1^ Bone Marrow Transplantation Center, Department of Hematology, The First Affiliated Hospital, School of Medicine, Zhejiang University, Hangzhou, China; ^2^ Institute of Hematology, Zhejiang University, Hangzhou, China; ^3^ Zhejiang Laboratory for Systems & Precision Medicine, Zhejiang University Medical Center, Hangzhou, China

**Keywords:** 1q gain, myeloma, bortezomib, chinese population, single center

## Abstract

**Background:**

Multiple myeloma is genetically heterogeneous, and chromosome abnormalities play a pivotal role in prognosis. A gain in chromosome 1q (+1q) is among the most common cytogenetic abnormalities; however, its relationship with overall survival (OS) and progression-free survival (PFS) in patients with multiple myeloma is still unclear. We aim to clarify the impact of +1q on the clinical characteristics and survival outcomes of patients treated with bortezomib-based combination regimes.

**Materials and methods:**

We retrospectively analyzed 258 patients first diagnosed with myeloma who underwent bortezomib-based therapy at the bone marrow transplantation department of a multiple myeloma treatment center in the first affiliated hospital of Zhejiang University, China.

**Results:**

We identified 258 newly diagnosed patients with multiple myeloma in our department from July 2013 to September 2018. We observed that 127 (49.2%) of the patients acquired +1q at diagnosis, and +1q strongly correlated with the occurrence of del(13q) and IgH rearrangement (*P* < 0.001). In the patients with +1q, the PFS was 22.2 months (95% CI 15.8–28.5 months), and the three-year and five-year PFS was 35.1% and 15.3%, respectively. Univariate analysis revealed that albumin, lactate dehydrogenase (LDH), and the percentage of plasma cells significantly affected PFS. Multivariate analysis showed that LDH and the percentage of plasma cells significantly affected PFS in the +1q patients. In terms of OS, the median OS for the +1q patients was 47.4 months (95% CI 34.7–59.5), while the OS of the non-+1q patients was not reached (*P* = 0.048). The univariate and multivariate analyses revealed that age, platelet count, and extramedullary lesions were significant adverse factors for OS in the +1q patients. There were no statistical differences between PFS and OS when there were other chromosomal abnormalities, but there was a decreased tendency in PFS. LDH and +1q also had a synergistic adverse effect on survival.

**Conclusion:**

+1q is associated with a higher tumor burden and correlated with the occurrence of del(13q) and IgH rearrangement at diagnosis. In the era of novel agents, +1q still significantly affects PFS and OS.

## Introduction

Multiple myeloma (MM) is the second most common hematologic malignancy derived from neoplastic plasma cells. It manifests with anemia, bone destruction, hypercalcemia, and renal dysfunction due to the monoclonal protein secreted by myeloma cells (1). Myeloma is genetically and biologically heterogeneous (2), with clinical characteristics that vary from person to person and are determined mainly by gene alterations, chromosomal abnormalities, tumor burdens, and numerous other biological factors. First presented in 1975, the Durie-Salmon staging system relies on parameters that predict tumor burden and is applied worldwide to evaluate disease outcomes. The subsequent international staging system (ISS), based on clinical data from patients who agreed to classical chemotherapy, has been widely adopted by hematologists (1, 3). However, both the Durie-Salmon staging system and ISS neglect the genetic abnormality of MM cells, which is a driver event in disease progression. Recurrent cytogenetic abnormalities are still the most potent predictors, classifying patients with MM into various risk levels and predicting their clinical outcomes. Based on cytogenetic abnormalities detected by fluorescence *in situ* hybridization (FISH), the revised ISS (R-ISS) incorporated t (4;14), t (14;16), and del17p, along with LDH and ISS, and is currently the standard MM staging system (4). In the era of target therapy and novel drugs, nearly all patients with MM benefit from proteasome inhibitors and immune modulators. However, disease progression is inevitable, even in low-risk patients. There could therefore be certain essential high-risk factors that have been overlooked (5).

FISH detection has revealed that chromosome 1 abnormalities are the most frequent cytogenetic disorders in patients with MM. Studies have reported that 35%–45% of patients with MM in western countries had chromosome 1 abnormalities. Moreover, the disorder frequency in Chinese individuals is as high as 70%, which might be due to differing disease stages among patients, and the settled threshold values differ from laboratories (6). To date, the clinical significance of 1q abnormalities remains controversial; however, significant research has revealed that 1q abnormalities are closely related to early disease progression and anti-myeloma resistance (7). The mSMART staging system of the Mayo Clinic ascribes the acquisition of 1q abnormalities to high-risk factors, but the system’s predictive value remains elusive.

Our study analyzed the clinical characteristics of patients who acquired 1q abnormalities and underwent bortezomib-based regimens in the department of bone marrow transplantation of the MM treatment center, in the first affiliated hospital of Zhejiang University, China, from 2013 to 2018 to elucidate the impact of 1q abnormalities on treatment efficacy and patient survival in our center in the era of novel MM drugs.

## Methods

### Patients and demographics

We performed a retrospective study of patients first diagnosed with MM in our center from July 2013 to September 2018. According to the World Health Organization and International Myeloma Working Group (IMWG) criteria, all patients were diagnosed and underwent at least one bortezomib-based therapy cycle and a thorough evaluation. All patients could be evaluated through inpatient/outpatient data or by phone from the start of the treatment to the end of the study.

### Treatment regimens

All patients underwent bortezomib and dexamethasone (PD)-based regimens. Based on the PD regimen, several patients received an additional drug, including cyclophosphamide, adriamycin, or thalidomide, as discussed below. After 3–4 cycles of induction therapy, the patients were divided into the autologous stem cell transplantation (ASCT) and non-ASCT groups based on the patients’ age, physical status, and willingness to undergo ASCT. All of the patients underwent maintenance therapy based on bortezomib, lenalidomide, or thalidomide.

### Clinical data acquisition

We collected the patient demographics and clinical data using our hospital’s inpatient software during the treatment. Laboratory indices included full blood count, liver and renal function, lactate dehydrogenase (LDH), bone marrow aspiration and biopsy, β2-microglobulin, blood and urine M spikes, and immune-fixation electrophoresis. We also performed computer tomography and magnetic resonance imaging to evaluate bone lesions and extramedullary invasion.

### Fluorescent *in situ* hybridization

We performed bone marrow aspiration before the initial treatment, acquiring 8-10 ml of heparinized bone marrow samples and separating CD138-positive cells by magnetic cell sorting according to the Miltenyi Biotec protocol. This study used 17p13, 1q21, 13q14, and 14q32 as target FISH probes. After probe hybridization, we counted 400 metaphase cells under fluorescence microscopy and defined the chromosome abnormalities according to **Table 1**. The cut-off for a positive test was 10% for 14q32 rearrangement and 20% for 1q gain, del(13q), and del(17p).

**Table 1:** Chromosome abnormalities of myeloma.

+1q: A gain of extra copies of 1q. Due to technological limitations, we did not perform the copy number confirmation of 1q amplification.
Del(13q): Fewer than two copies of 13q.
Del(17p): Fewer than two copies of 17p.
14q32 rearrangement: Detailed translocation was identified, including t(4;14), t(11;14) and t(14;16).

### Stage assessment

Before treatment, we thoroughly assessed all patients using the laboratory results and disease history. We then assigned the patients to the various clinical stages according to the Durie-Salmon staging system and ISS (8). Our study retrospectively reconfirmed the patients’ initial disease stage. Due to the lack of detailed IgH translocation information, R-ISS was not performed.

### Response criteria to anti-myeloma therapy

We applied the IMWG response criteria to evaluate the treatment efficacy. The standards divided patients into complete remission, very good partial remission, partial remission, stable disease, and disease progression (3). We followed up the patients from the first treatment cycle to disease progression or death due to any cause. Progression-free survival (PFS) was the time from the initiation of therapy to MM progression, death, or the end of follow-up. Overall survival (OS) was the time from the initiation of therapy to death or the end of follow-up.

### Statistical analysis

We followed up on all patients until June 30, 2019, and evaluated each treatment cycle. The significance analysis of the continuous variables employed an independent sample t-test. The categorical variables were compared using the Pearson chi-squared test or Fisher’s exact test. We estimated the PFS and OS using the Kaplan-Meier method and the log-rank test to compare them between the groups. We also used the Cox proportional hazards model to identify PFS and OS factors. A 2-tailed P-value <0.05 was significant for all tests, and P-values <0.2 can be estimated using multiple variables. We used SPSS v.26.0 for Windows for all the statistical tests.

## Results

### Patient characteristics

Our study identified 258 patients newly diagnosed with MM who underwent bortezomib-based therapy in our center. The patients’ median age was 62 years (range 31–84 years), with 146 male and 112 female patients. According to the FISH test, 127 (49.2%) patients were positive for 1q gain at diagnosis. The clinical characteristics are summarized in **Table 2**. There was no statistically significant difference in +1q among the various ages, sex, type of M spike, and serum creatine levels. The occurrence of extramedullary lesions did not differ significantly between the +1q and 1q normal groups (23.6% vs. 26%). The patients with normal 1q had a lower Durie-Salmon stage (1A and 2A: 26% vs. 11%, *P* = 0.009) and a tendency for a lower ISS stage (38.2% vs. 28.3%, *P* = 0.153) and lower LDH levels (172 U/L vs. 193 U/L, *P* = 0.113). The patients with normal 1q also had higher median hemoglobulin levels (99.5 g/L vs. 87 g/L, *P* = 0.001) and higher platelets levels (190*10e9/L vs 169.5*10e9/L, *P* = 0.320). Additionally, the patients with +1q at diagnosis showed a higher percentage of neoplastic plasma cells (31.0% vs. 19.5%, *P* = 0.001).

**Table 2:** Baseline clinical characteristics and 1q abnormalities of the patients with multiple myeloma.

Patients’ clinical characteristics	All (N = 258)	1q normal (n = 131)	1q gain (n = 127)	*P*
Sex(male) n (%)	146 (56.6)	72 (55.0)	74 (58.3)	0.592
Age(≤65 y), n (%)	156 (60.5)	85 (64.9)	71 (55.9)	0.140
Type of M protein, n (%)				0.413
IgA	63 (24.4)	29 (22.1)	34 (26.8)	
IgG	123 (47.7)	60 (45.8)	63 (49.6)	
IgD	15 (5.8)	8 (6.1)	7 (5.5)	
Light chain	55 (21.3)	32 (24.4)	23 (18.1)	
Double clone	2	2 (1.5)	0 (0)	
Durie-Salmon staging system, n (%)				0.009
1A+2A	48 (18.6)	34 (26.0)	14 (11.0)	
3A	167 (64.7)	77 (58.7)	90 (70.9)	
2B+3B	43 (16.7)	20 (15.3)	23 (18.1)	
ISS staging system				0.153
1	86 (33.3)	50 (38.2)	36 (28.3)	
2	79 (30.6)	34 (26.0)	45 (35.4)	
3	93 (36.0)	47 (35.9)	46 (36.1)	
Hb (g/L), median (range)	95.0 (42.0–161.0)	99.5 (51.0–161.0)	87.0 (42.0–142.0)	0.001
platelet (×10e9/L), median (range)	177 (23–513)	190 (42–513)	169.5 (23–397)	0.032
Creatine (umol/L), median (range)	84 (33–1033)	87 (33–945)	82 (39–1033)	0.864
LDH (u/L), median (range)	185 (79–5758)	172 (79–550)	193 (94–5758)	0.113
Percentage of bone marrow plasma cells (%) median (range)	29.0 (3.0–99.0)	19.5 (3.0–99.0)	31.0 (4.5–97.0)	0.001
Calcium, median (range)	2.25 (1.62–3.95)	2.26 (1.78–3.95)	2.24 (1.62–3.93)	0.728
CRP, median (range)	2.25 (0–204.2)	2.4 (0–204.2)	2.0 (0–192.2)	0.649
Alb, median (range)	38.4 (19.5–64.3)	38.6 (19.5–53.0)	37.8 (21.3–64.3)	0.234
β2MG, median (range)	3.90 (1.03–40.1)	3.64 (1.03–40.1)	4.10 (1.08–30.61)	0.650
Extramedullary lesions, n (%)	64 (24.8)	34 (26.0)	30 (23.6)	0.665
Therapy, n (%)				0.176
PAD	34 (13.2)	21 (16.0)	13 (10.2)	
PCD	159 (61.6)	82 (62.6)	77 (60.6)	
PTD	17 (6.6)	5 (3.8)	12 (9.4)	
PD	48 (18.6)	23 (17.6)	25 (19.7)	
ASCT, n (%)	33 (12.8)	18(13.7)	15 (11.8)	0.643
Del (13q), n (%)	111 (43.0)	35 (26.7)	76 (59.8)	<0.001
Del (17p), n (%)	14 (5.4)	5 (3.8)	9 (7.1)	0.246
IgH rearrangement, n (%)	86 (33.3)	25 (19.1)	61 (48.0)	<0.001
FISH abnormalities (excluding 1q), n (%)				<0.001
0	116 (45.0)	82 (62.6)	34 (26.8)	
1	82 (31.8)	35 (26.7)	47 (37.0)	
2–3	60 (23.2)	14 (10.7)	46 (36.2)	

### Relationship between +1q and other chromosomal abnormalities

Among the included patients, +1q was strongly correlated with the occurrence of del(13q) and IgH rearrangement (*P* < 0.001) (**Table 2**). Of the patients who acquired +1q, 59.8% (76/127) had co-occurring del(13q), while only 26.7% (35/127) of those without 1q gain had del(13q). The same as del(13q), 48% (61/127) co-occurred IgH rearrangement with +1q, while only 19.1% (25/131) had IgH rearrangement in those without +1q. Previous studies have reported that various chromosomal abnormalities always co-occur. Our data indicated that the patients with +1q had a higher frequency of acquiring other accompanying chromosomal abnormalities than those without +1q, and the difference was statistically significant. In our study, 47 of the +1q patients (37%) had co-occurrence of one other abnormality, and 46 of the +1q patients (36.2%) had 2–3 chromosomal abnormalities. In contrast, 26.7% (35/131) of the patients without +1q acquired another FISH abnormality, and only 10.7% (14/131) had 2–3 chromosomal abnormalities co-occurrence.

### Survival analysis

**Figure 1:**
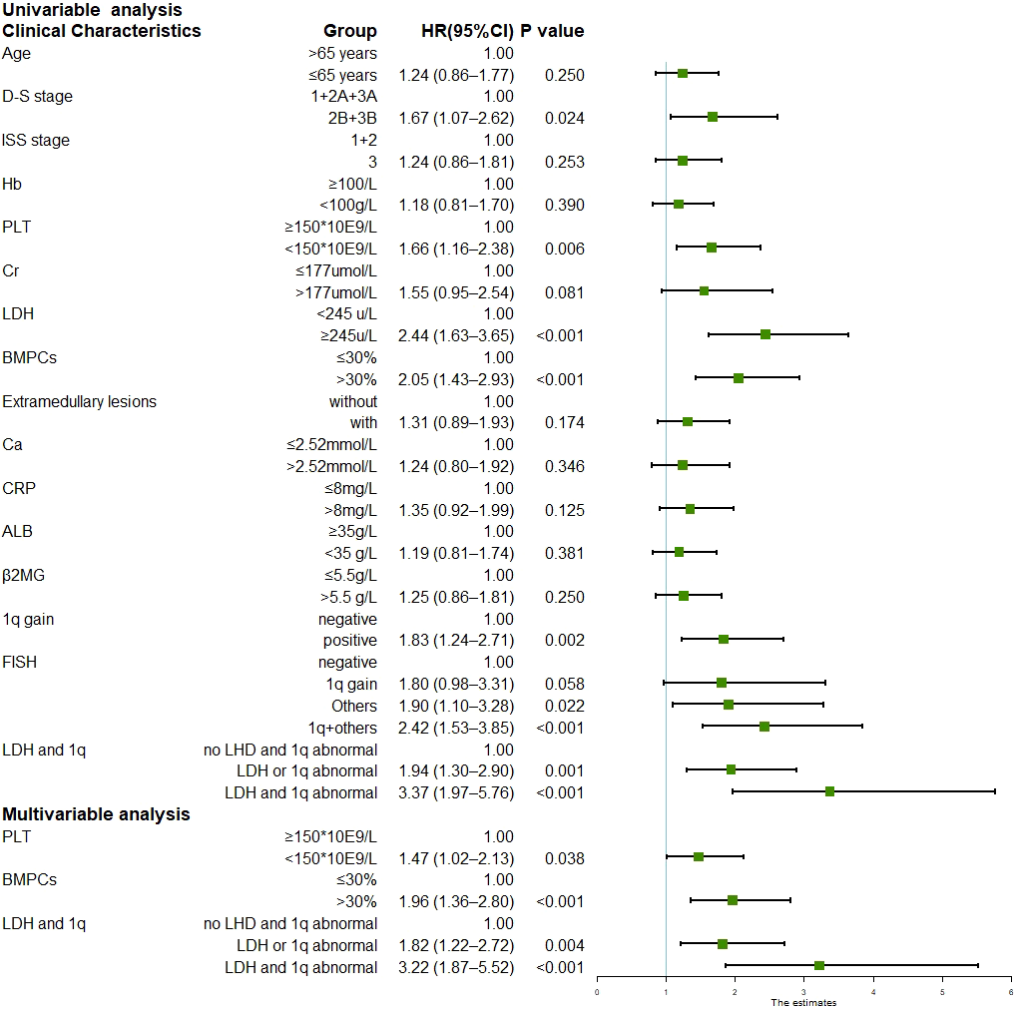
Univariable and multivariable analysis for progression-free survival (Data can be found in **Supplementary Table 2**).

**Figure 2:**
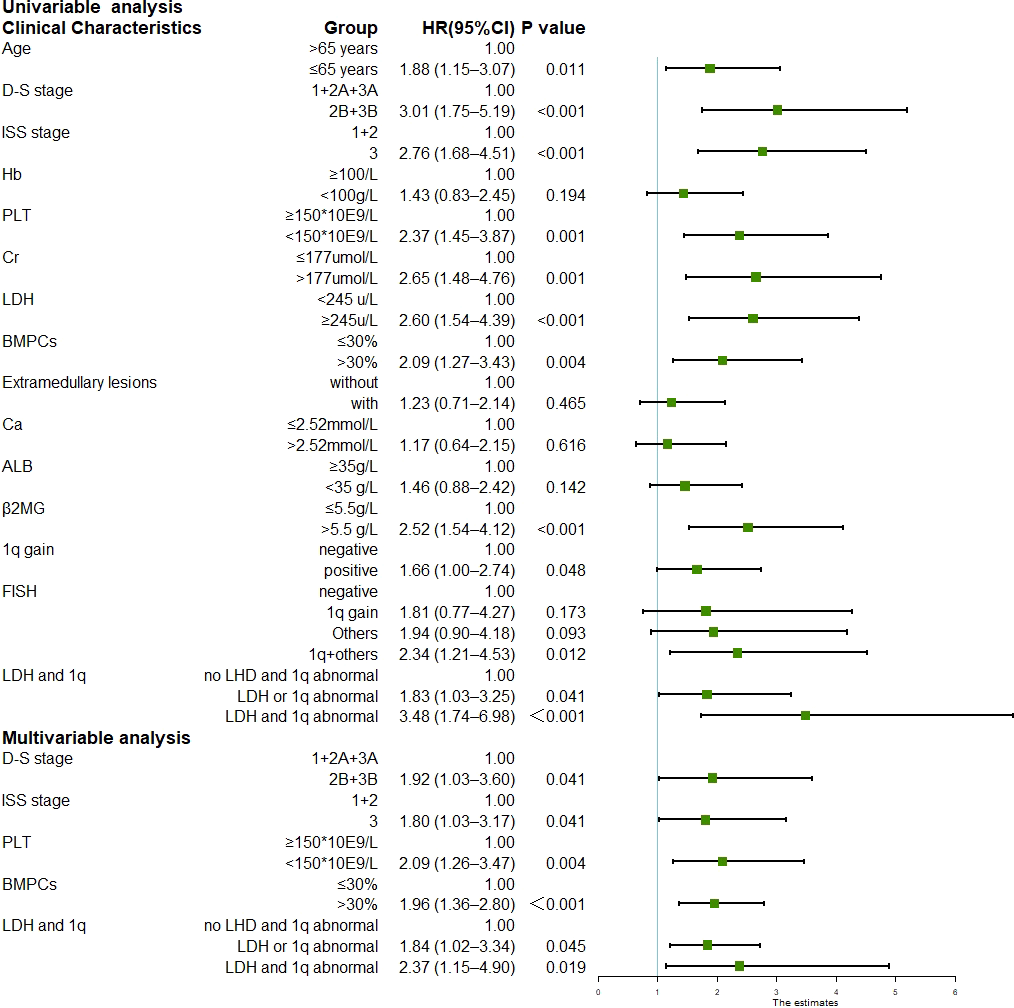
Univariable and multivariable analysis for overall survival (Data can be found in **Supplementary Table 3**).

The median follow-up duration was 24.1 months (range, 1.1–71.0 months) for the surviving patients. The median PFS was 29.6 months (95% CI 22.8–36.4), and the three-year and five-year PFS was 42.1% and 21.7%, respectively. The median OS was not reached; the three-year and five-year OS was 68.8% and 53.3%, respectively. Univariate analysis of all 258 patients revealed that the baseline factors Durie-Salmon staging, platelet count, LDH, bone marrow plasma cell percentage, FISH abnormality, and +1q were significantly associated with PFS (**Figure 1**). Age, Durie-Salmon staging, ISS staging, platelet count, LDH, C-reactive protein (CRP), bone marrow plasma cell percentage, and +1q significantly affected OS (**Figure 2**). Multivariate analysis showed that the platelet count, percentage of plasma cells, and an LDH greater than the upper normal limit (UNL) and/or +1q were adverse factors that affect PFS (**Figure 1**). The percentage of plasma cells, platelet counts, and LDH > UNL and +1q were all significant factors (*P* < 0.001). Durie-Salmon staging, ISS staging, platelet count, LDH > UNL and/or +1q had a significant adverse impact on OS in the multivariate analysis (**Figure 2**).

### Impact of +1q on survival

**Figure 3:**
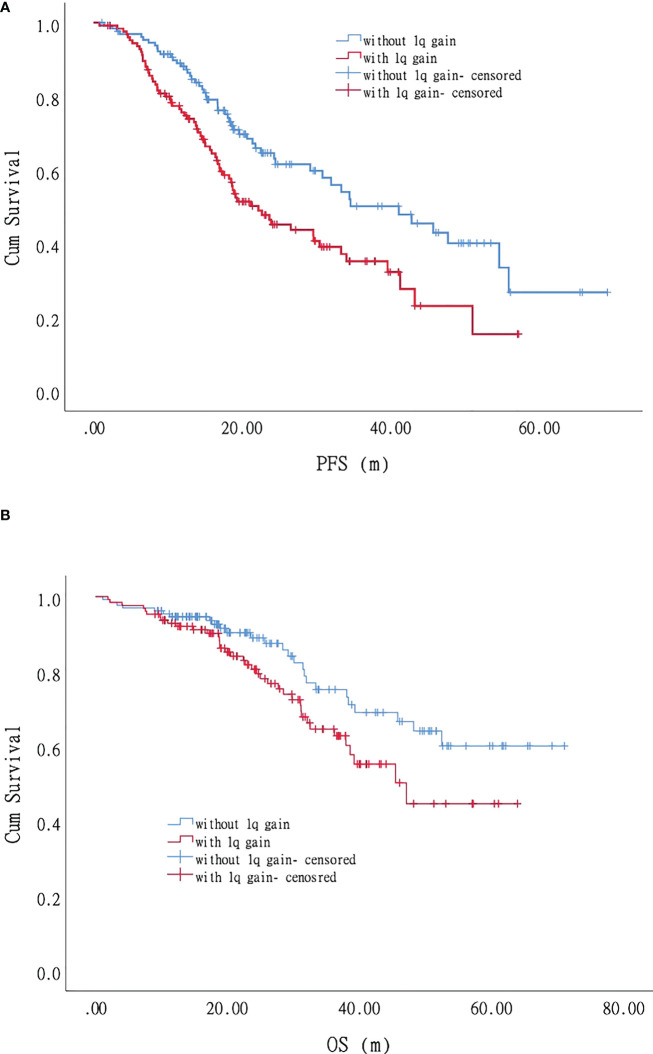
Impact of +1q on progression-free survival (PFS) and overall survival (OS) of patients with myeloma: Patients with +1q had shorter PFS and OS. **(A)**. Progression-free survival. **(B)**. Overall survival.

For the patients with +1q, the PFS was 22.2 months (95% CI 15.8–28.5), and the three-year and five-year PFS was 35.1% and 15.3%, respectively (**Supplementary Table 1**; **Figure 3A**). Univariate analysis on 127 patients who acquired +1q and 131 patients without +1q showed that albumin, LDH, and the percentage of plasma cells significantly affected PFS. For the patients without +1q, only LDH had an adverse impact on PFS. The multivariate analysis of 258 patients showed that LDH and the percentage of plasma cells significantly affected PFS in the +1q patients. For the patients without +1q, LDH, the percentage of plasma cells, and Durie-Salmon staging (2B+3B vs. 1-3A) played a pivotal role in PFS. For the patients without +1q, the PFS was 41.1 months (95% CI 28.6–53.6), and the three-year and five-year PFS was 51.3% and 26.7%, respectively, which were significantly longer than those for the +1q patients. Furthermore, a 1q gain also affected the OS of the patients with MM. Among the patients with +1q, the median OS was 47.4 months (95% CI 34.7–59.5), while the OS of the patients without +1q was not reached (*P* = 0.048). The three-year OS was 64.5% and 75.1% in the patients with +1q and those without +1q, respectively; the five-year OS was 44.6% and 60.0%, respectively (**Figure 3B**). Univariate analysis to evaluate the factors’ impact on OS revealed that age, platelet count, CRP, β-microglobulin, and extramedullary lesions were significant adverse factors for OS in the +1q patients. Durie-Salmon staging (2B+3B vs. 1-3A), ISS stage II and stage III, serum creatine, and LDH could significantly affect the OS of patients without +1q. The multivariate analysis showed that age, ISS stage III, platelet count, percentage of plasma cells, and extramedullary lesions were significant adverse factors for OS in +1q patients. In contrast, Durie-Salmon staging (2B+3B vs. 1-3A) and platelet count affected the OS of the patients without +1q.

**Figure 4:**
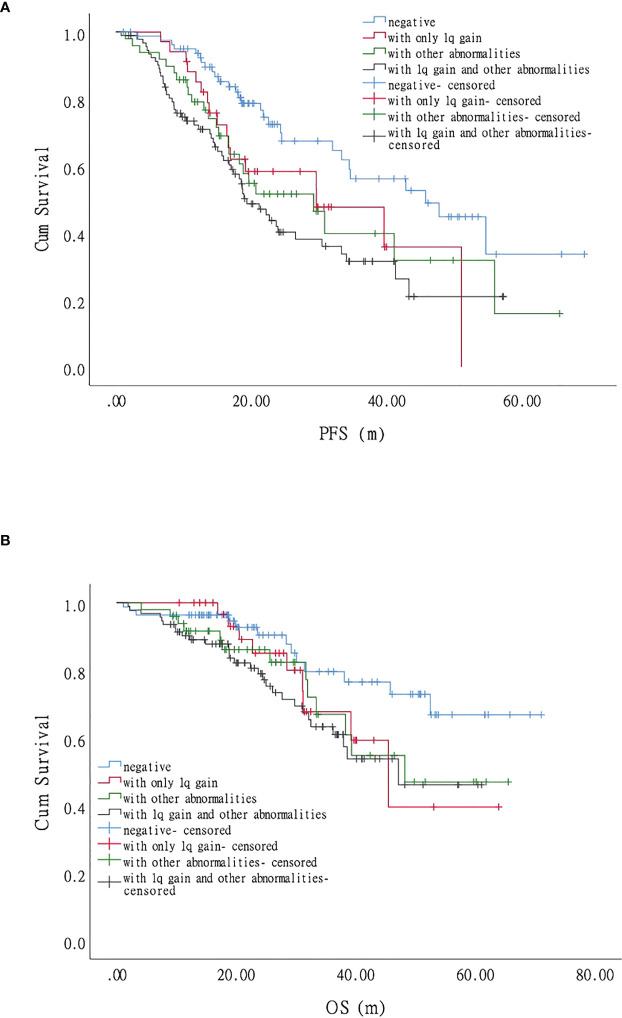
Impact of 1q gain and its accompanying chromosomal abnormalities on the survival of patients with myeloma. **(A)**. Progression-free survival (PFS) was significantly longer when comparing the FISH-negative group with the other abnormalities group and the +1q and other abnormalities group, respectively, but there was no significant difference with the +1q group. **(B)**. Compared with the fluorescence *in situ* hybridization (FISH)-negative group, only the other abnormality group, showed a significant difference in overall survival (OS).

Most patients with MM have more than one chromosomal abnormality. Our data showed that 127 patients acquired +1q, 73%(93/127)of whom had other accompanying chromosomal abnormalities. We, therefore, further divided the patients according to the FISH results into FISH negative, +1q, with other abnormalities (chromosomal abnormalities other than +1q), and with +1q and other abnormalities (chromosomal abnormalities accompanying +1q) and analyzed their survival conditions (**Supplementary Table 1**). Our data revealed that the median PFS for each group was 45.7 (95% CI 27.6–63.8), 29.6 (95% CI 11.9–47.3), 29.2 (95% CI 17.6–40.7), and 19.3 months (95% CI 15.3–23.3), respectively, showing statistically significant differences in PFS and OS when comparing the FISH negative group with the other three groups (*P* < 0.05) (**Figure 1**; **Figure 4A**). The hazard ratio was 1.8 (95% CI 1.0–3.3) for the +1q group, 1.9 (95% CI 1.1–3.3) for the other abnormalities group, and 2.4 (95% CI 1.5–3.8) for the +1q and other abnormalities group. The median OS was not reached in the FISH negative group, 45.4 months (95% CI 34.2–56.6) in the +1q group, 48.2 months (95% CI NE) in the other abnormalities group, and 47.1 months (95% CI NE) in the +1q with other abnormalities group. Compared with the FISH-negative group, only the group with other abnormalities significantly differed. The hazard ratio was 1.81 (95% CI 0.77–4.27) for the +1q group, 1.9 (95% CI 0.90–4.18) for the other abnormalities group, and 2.34 (95% CI 1.21–4.53) for the group with +1q and other abnormalities (**Figure 2**; **Figure 4B**).

### Impact of lactate dehydrase on survival

**Figure 5:**
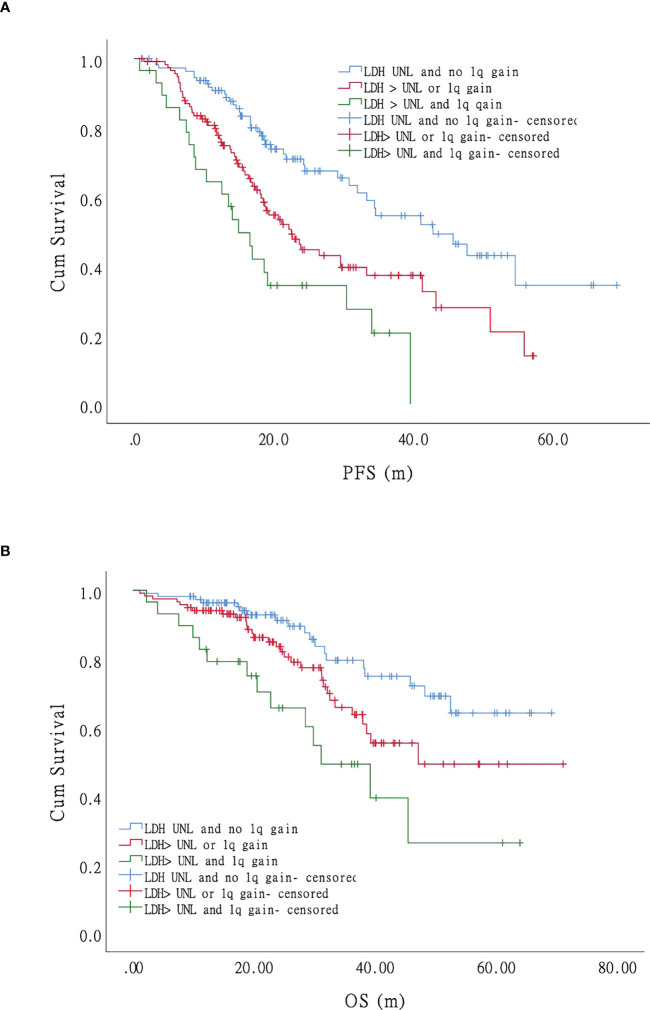
Impact of LDH on progression-free survival (PFS) and overall survival (OS) of patients with myeloma: LDH and 1q gain have an adverse effect on the survival (both PFS and OS) of MM patients independently. Patients with co-occurrence of LDH>UNL and 1q gain (the green curve) had the worst survival while those without LDH nor 1q abnormal (the blue curve) had the most favorable survival. Patients with LDH>UNL or 1q gain (the red curve) showed intermediate compared with patients mentioned above. **(A)**. Progression-free survival. **(B)**. Overall survival.

In the univariate analysis, only LDH independently affected both PFS and OS significantly, indicating that LDH plays a crucial role in MM survival. We performed further univariate tests for PFS and OS. We divided the patients into three groups: negative (no LDH > UNL and +1q), single-positive (LDH > UNL or +1q), and double-positive (LDH > UNL and +1q). There were statistically significant differences in the PFS of the three groups (**Figure 5A**). The hazard ratio was 1.94 (95% CI 1.30–2.90) for the single-positive group and 3.37 (95% CI 1.97–5.76) for the double-positive group. The OS was significantly lower in the double-positive group when compared with the negative group, and the hazard ratio was 3.48 (95% CI 1.74–6.98) in the +1q and LDH > UNL group (**Figure 5B**).

## Discussion

In this study, we retrospectively observed the impact of the +1q abnormality on the survival and prognosis of patients with MM based on proteasome inhibitor (PI) therapy. In the era of new MM drugs, PI is the cornerstone of myeloma treatment and is widely applied in a variety of regimes, including PTD (bortezomib, thalidomide, and dexamethasone), PCD (bortezomib, cyclophosphamide, and dexamethasone) and PRD (bortezomib, lenalidomide, and dexamethasone). Until recently, +1q has been thought to have no significant impact on OS and PFS. With the emergence of new drugs such as PI and lenalidomide, however, the significance of +1q has changed (9). In 2022, the European Myeloma Network, published the second revision of the international staging system, which analyzed the value of each risk feature, including +1q. In the study, authors assigned +1q 0.5 points, stratified patients into four risk groups and predicted patients’ survival more accurately (10). Our study revealed that +1q occurred in approximately 49.2% of the 258 patients, a higher rate than in western countries (approximately 35%–45%). Compared with the patients without +1q, the +1q patients had more advanced Durie-Salmon stages, lower hemoglobin levels, and a higher percentage of MM cells in the bone marrow. Studies have also reported that +1q is associated with decreased platelet counts, increased beta-globulin levels, and more advanced ISS stages (9).

Recurrent chromosomal abnormalities play a pivotal role in myeloma pathogenesis and have major prognostic value in disease management. +1q is one of the most important chromosomal changes in differing stages of myeloma. From monoclonal gammopathy of undetermined significance, smoldering multiple myeloma, and newly diagnosed MM to relapsed and refractory multiple myeloma, the rate of +1q increases, which implies a relationship between +1q and disease progression (11), which might be due to the derangement of genes located on 1q21, including MCL-1, IL-6R, and CKS1B, all of which are closely related to myeloma prognosis (11, 12). The impact of +1q on the prognosis is still unclear and has not been incorporated into the high-risk factors in most guidelines. A previous study reported that patients could be classified into a high-risk group only when they were defined as ISS III and with 1q amplification of more than three copies (13). Moreover, our study and previous research showed that +1q is closely associated with t(4;14) and t(14;16), which are relevant to IgH rearrangement, and del(17p) had no connection to +1q (9, 14).

As with our study, other studies have reported that patients with +1q have shorter survival times in survival analyses, both in PFS and OS. PFS was significantly shorter when other adverse factors accompanied +1q (9, 15). In the UK myeloma IX and myeloma XI studies, 1q21-positive patients had shorter PFS and OS. When analyzed with multivariate parameters, they were thought to be an independent adverse prognostic factor, with advanced ISS stage and translocation associated with IgH rearrangement. OS showed no significant difference between the +1q patients and those with amplification; however, the patients with 1q amplification had a shorter PFS (15). However, our study observed no impact of +1q on PFS (29.6 vs. 29.2 months) compared with other adverse FISH factors, which might be because the studies mentioned above detected the copy number of 1q abnormalities, which we did not.

Given that the patients in our analysis underwent bortezomib-based therapy, the OS and PFS in this study reflected the efficacy of bortezomib in overcoming the adverse impact of +1q. Despite being administered bortezomib and dexamethasone combined with thalidomide, cyclophosphamide, or adriamycin in three-drug or two-drug regimes, the patients with +1q still had shorter survival times. A retrospective study from the Mayo Clinic showed that +1q was an adverse factor regardless of whether the patients underwent PI, an immunomodulatory drug, or PI plus immunomodulatory drug regimes (9, 16). Interestingly, Schmidt reported that patients diagnosed with MM with +1q had a better response when treated with the PRD regime than those without +1q; however, the former still had poor prognoses (14, 17). In our analysis, +1q was an adverse prognostic factor and induced a shorter survival time even in the era of new MM drugs.

ASCT is an essential part of myeloma treatment. Varma et al. showed that +1q was an independent factor affecting the OS of patients with myeloma who previously underwent bortezomib-based regimes and then underwent ASCT. For three years, the PFS and OS were 41% and 79% for the +1q patients, respectively, while in the compared group, these increased to 56% and 86%, respectively. The patients with +1q had a more significant risk of death and progression (hazard ratio 2.21, CI 1.18–4.16, P = 0.014). The median PFS was 26.6 months for the +1q patients and 38.8 months for the patients without +1q (HR 1.9, CI 0.9–4.08, P = 0.09) (18). A recent multicenter study in China reported that in patients who were administered novel agents included in regimes followed by ASCT, the isolated gain of 1q21 was an independent adverse predictor of PFS (35 vs. 66 months) and OS (61 vs. 100 months). This study indicated that 1q21 gain could independently predict shorter PFS and OS (19).

In our study, we mainly focus on 1q gain in NDMM patients, however, refractory/relapsed patients (RRMM) also deserved to investigate. As we know, gene mutations in RRMM patients are relatively complex. Multiple attacks act on chromosomes, finally driving patients to become refractory or relapse. Thus, the relationship between cytogenetic abnormality and novel therapies is worth exploring. Patients refractory to PIs, IMiDs, and mAbs are defined as triple-class refractory (TCR) MM. A recent study reported that high-risk cytogenetic features, including t(4;14), t(14;16), t(14;20), deletion 17p, TP53 mutation, and 1q gain, are associated with a shorter time to develop to TCR status (2.9 years vs. 2.3 years). Moreover, when patients progressed to TCR status, 31% of patients got a new 1q duplication change (10). Chimeric antigen receptor (CAR)-T cell therapy is quite promising in the field of myeloma treatment, usually applied in refractory/relapsed myeloma (RRMM) patients. B cell maturation antigen is the commonest target of myeloma CAR-T therapy (20). Recent clinical trials showed that the complete remission rate varies from 33% to 76.5% in RRMM patients (21, 22).Zhang et al. explored the risk factors of RRMM patients who received CAR-BCMA T cell therapy, and their results showed 1q gain had an adverse impact on survival (22).

Our study has certain limitations, including its single-center, retrospective nature. We did not analyze the copy number of 1q amplification in detail and did not explore the impact of +1q on ASCT, CD38 monoclonal antibodies, and chimeric antigen receptor T-cell therapy.

## Data availability statement

The original contributions presented in the study are included in the article/**Supplementary Material**. Further inquiries can be directed to the corresponding authors.

## Author contributions

ZC and JH designed the study. QC and JH performed the statistic work and wrote the article. The rest of the authors collected data of this paper. All authors contributed to the article and approved the submitted version.
