# Supplementary material for: The adverse impact of a gain in chromosome 1q on the prognosis of multiple myeloma treated with bortezomib-based regimens: A retrospective single-center study in China
Source: Front Oncol. 2022 Dec 20;12:1084683. doi: 10.3389/fonc.2022.1084683 (PMC9808074; doi:10.3389/fonc.2022.1084683)
Supplement: Supplementary file 1 [file Table_1.pdf]

Supplementary Table 1 Survival of patients with multiple myeloma

Median Survival, m (95% CI)	PFS	P	OS	P
Age ≤65 (n = 156)	32.0 (23.7–40.3)	0.249	NR	0.011
＞65 (n = 102)	21.2 (12.7–29.7)		45.4 (33.9–56.9)	
D-S 1-3A (n = 215)	40.4 (25.8–38.2)	0.023	NR	<0.001
2B+3B (n = 43)	17.7 (15.1–20.4)		33.4 (21.3–45.4)	
ISS 1+2 (n = 165)	32.0 (27.8–36.1)	0.252	NR	<0.001
3 (n = 93)	19.1 (10.0–28.3)		36.2 (27.2–45.2)	
Hgb ＜100 g/L (n = 150)	29.6 (20.4–38.7)	0.389	NR	0.192
≥100 g/L (n = 108)	33.3 (21.0–45.6)		52.4	
Plt ＜150 × 10e9/L (n = 164)	21.8 (11.8–31.7)	0.006	NR	<0.001
≥150 × 10e9/L (n = 92)	33.3 (21.1–45.5)		45.8 (29.0–62.6)	
Cr ≤177 umol/L (n = 220)	30.8 (23.2–38.4)	0.078	NR	0.001
＞177 umol/L (n = 38)	18.6 (13.9–23.3)		39.2 (28.2–50.2)	
LDH ≤ UNL (n = 209)	33.3 (24.5–42.2)	<0.001	NR	<0.001
＞UNL (n = 49)	16.6 (11.9–21.3)		33.4 (23.0–43.8)	
Plasma cells ≤30% (n = 149)	34.4 (20.9–48.0)	<0.001	NR	0.003
＞30% (n = 109)	18.2 (12.0–24.4)		45.4 (35.5–55.3)	
Extramedullary lesion negative (n = 194)	30.4 (23.2–37.7)	0.174	NR	0.464
positive (n = 64)	21.8 (5.4–38.1)		NR	
Ca ≤ UNL (n = 215)	30.8 (23.4–38.2)	0.346	NR	0.616
＞UNL (n = 43)	23.9 (11.1–36.7)		NR	
CRP ≤ UNL (n = 191)	30.8 (23.1–38.5)	0.125	NR	0.022
＞UNL (n = 67)	22.7 (9.0–36.4)		45.8 (31.3–60.2)	
Alb ≥ UNL (n = 179)	30.8 (22.7–38.9)	0.382	NR	0.142
＜ UNL (n = 79)	24.5 (10.6–38.4)		39.1	
β2 MG ≤ 5.5 (n = 167)	32.0 (27.6–36.3)	0.250	NR	<0.001
＞ 5.5 (n = 91)	19.3 (10.7–28.0)		38.6 (24.2–52.9)	
1q gain negative (n = 131)	41.1 (28.6–53.6)	0.002	NR	0.048
positive (n = 127)	22.2 (15.8–28.5)		47.4 (34.7–59.5)	
FISH negative (n = 82)	45.7 (27.6–63.8)	0.002	NR	0.081
1q gain (n = 34)	29.6 (11.9–47.3)		45.4 (34.2–56.6)	
Others (n = 49)	29.2 (17.6–40.7)		48.2(NE)	
1q+others (n = 93)	19.3 (15.3–23.3)		47.1(NE)	
LDH and 1q		<0.001		0.001
no LHD and 1q abnormal (n = 111)	42.8 (30.0–55.6)		NR	
LDH or 1q abnormal (n = 118)	22.5 (18.0–27.1)		47.1	
LDH and 1q abnormal (n = 29)	15.0 (10.8–19.1)		31.1 (20.1–42.0)	

UNL: Upper normal limit

Supplementary Table 2 Univariable and multivariable analysis for progression-free survival

Univariable analysis			Multivariable analysis		
Variable	HR (95% CI)	P	Variable	HR (95% CI)	P
Age ≥65 y	1.24 (0.86–1.77)	0.250	Plt ＜150 × 10e9/L	1.47 (1.02–2.13)	0.038
D-S 1–3A vs. 2B+3B	1.67 (1.07–2.62)	0.024	Plasma cells ＞30%	1.96 (1.36–2.80)	＜0.001
ISS 1+2 vs. 3	1.24 (0.86–1.81)	0.253	LDH and 1q		
Hgb ＜100 g/L	1.18 (0.81–1.70)	0.390	no LHD and 1q abnormal	1	＜0.001
Plt ＜150 × 10e9/L	1.66 (1.16–2.38)	0.006	LDH or 1q abnormal	1.82 (1.22–2.72)	0.004
Cr ＞177 umol/L	1.55 (0.95–2.54)	0.081	LDH and 1q abnormal	3.22 (1.87–5.52)	＜0.001
LDH ＞UNL	2.44 (1.63–3.65)	＜0.001			
Plasma cells ＞30%	2.05 (1.43–2.93)	＜0.001			
Extramedullary lesions	1.31 (0.89–1.93)	0.174			
Ca ＞UNL	1.24 (0.80–1.92)	0.346			
CRP ＞UNL	1.35 (0.92–1.99)	0.125			
Alb ＜35 g/L	1.19 (0.81–1.74)	0.381			
β2MG ＞5.5 g/L	1.25 (0.86–1.81)	0.250			
1q gain positive	1.83 (1.24–2.71)	0.002			
FISH negative	1	0.003			
1q gain	1.80 (0.98–3.31)	0.058			
Others	1.90 (1.10–3.28)	0.022			
1q+others	2.42 (1.53–3.85)	＜0.001			
LDH and 1q					
no LHD and 1q abnormal	1	＜0.001			
LDH or 1q abnormal	1.94 (1.30–2.90)	0.001			
LDH and 1q abnormal	3.37 (1.97–5.76)	＜0.001			

UNL: Upper normal limit

Supplementary Table 3 Univariable and multivariable analysis for overall survival

Univariable analysis			Multivariable analysis		
Variable	HR (95% CI)	P	Variable	HR (95% CI)	P
Age ≥65 y	1.88 (1.15–3.07)	0.011	D-S 2B+3B vs. 1-3A	1.92 (1.03–3.60)	0.041
D-S 2B+3B vs. 1-3A	3.01 (1.75–5.19)	<0.001	ISS 3 vs 1+2	1.80 (1.03–3.17)	0.041
ISS 3 vs 1+2	2.76 (1.68–4.51)	<0.001	Plt <150 × 10e9/L	2.09 (1.26–3.47)	0.004
Hgb <100 g/L	1.43 (0.83–2.45)	0.194	LDH and 1q		
Plt <150 × 10e9/L	2.37 (1.45–3.87)	0.001	no LHD and 1q abnormal	1	0.042
Cr >177 umol/L	2.65 (1.48–4.76)	0.001	LDH or 1q abnormal	1.84 (1.02–3.34)	0.045
LDH > UNL	2.60 (1.54–4.39)	<0.001	LDH and 1q abnormal	2.37 (1.15–4.90)	0.019
Plasma cells >30%	2.09 (1.27–3.43)	0.004			
Extramedullary lesions	1.23 (0.71-2.14)	0.465			
Ca > UNL	1.17 (0.64–2.15)	0.616			
Alb <35 g/L	1.46 (0.88–2.42)	0.142			
β2MG >5.5 g/L	2.52 (1.54–4.12)	<0.001			
1q gain positive	1.66 (1.00–2.74)	0.048			
FISH negative	1	0.092			
1q gain	1.81 (0.77–4.27)	0.173			
Others	1.94 (0.90–4.18)	0.093			
1q+others	2.34 (1.21–4.53)	0.012			
LDH and 1q					
no LHD and 1q abnormal	1	0.002			
LDH or 1q abnormal	1.83 (1.03–3.25)	0.041			
LDH and 1q abnormal	3.48 (1.74–6.98)	<0.001			

UNL: Upper normal limit
